# A systematic review on sensor-based driver behaviour studies: coherent taxonomy, motivations, challenges, recommendations, substantial analysis and future directions

**DOI:** 10.7717/peerj-cs.632

**Published:** 2021-08-25

**Authors:** Ward Ahmed Al-Hussein, Miss Laiha Mat Kiah, Por Lip Yee, B B. Zaidan

**Affiliations:** 1 Department of Computer System and Technology, Faculty of Computer Science and Information Technology, University of Malaya, Kuala Lumpur, Malaysia; 2 Department of Computing, Faculty of Arts, Universiti Pendidikan Sultan Idris, Perak, Malaysia

**Keywords:** Driver behaviour, Sensors, ADAS, Intelligent transportation systems, Naturalistic driving, Traffic safety

## Abstract

In the plan and development of Intelligent Transportation Systems (ITS), understanding drivers behaviour is considered highly valuable. Reckless driving, incompetent preventive measures, and the reliance on slow and incompetent assistance systems are attributed to the increasing rates of traffic accidents. This survey aims to review and scrutinize the literature related to sensor-based driver behaviour domain and to answer questions that are not covered so far by existing reviews. It covers the factors that are required in improving the understanding of various appropriate characteristics of this domain and outlines the common incentives, open confrontations, and imminent commendations from former researchers. Systematic scanning of the literature, from January 2014 to December 2020, mainly from four main databases, namely, IEEEXplore, ScienceDirect, Scopus and Web of Science to locate highly credible peer-reviewed articles. Amongst the 5,962 articles found, a total of 83 articles are selected based on the author’s predefined inclusion and exclusion criteria. Then, a taxonomy of existing literature is presented to recognize the various aspects of this relevant research area. Common issues, motivations, and recommendations of previous studies are identified and discussed. Moreover, substantial analysis is performed to identify gaps and weaknesses in current literature and guide future researchers into planning their experiments appropriately. Finally, future directions are provided for researchers interested in driver profiling and recognition. This survey is expected to aid in emphasizing existing research prospects and create further research directions in the near future.

## Introduction

Traffic accidents are one of the main sources of injuries in the 21st century (Carmona et al., 2015). Internationally, road accidents are considered the third widespread cause of death next to cardiovascular diseases and cancer (Foo, 2015). In the United States alone, traffic crashes cause 30,000 to 40,000 fatalities annually and are the number one cause of death among the 8 to 24 years old (Guo, 2019). The majority of accidents are ascribed to human-related factors, such as inclination towards aggressive driving (Demir, Demir & Özkan, 2016). Understanding the behaviour of drivers during certain events on the road is important to reduce accidents rate and improve traffic congestions (Derbel, 2018).

There are several methods to acquire driving data from drivers, such as the use of simulations, questionnaires, observations, and crash reports. However, most of these methods are criticized in the literature for being biased (Ziakopoulos et al., 2020). To solve this issue, researchers aim to collect driving data from drivers in real-time experiments, using sensors installed inside their vehicles. Studies that collect driving data from such experimental settings are usually referred to as naturalistic driving studies (NDS) (Cai et al., 2018). Some researchers utilize an existing NDS dataset, that is available online, in their studies (Chen, Kusano & Gabler, 2015), while others collect data derived from in-vehicle sensors (Park et al., 2016), thus producing their datasets.

The current literature lacks guidelines on how to plan experiments fittingly for data collection. This is because existing surveys that covered data collection using in-vehicle sensors have few limitations of their own. They frequently review a specific area in the driver behaviour domain, such as car-following behaviour (Saifuzzaman & Zheng, 2014), lane changing behaviour (Koesdwiady et al., 2016), intersection behaviour (Shirazi & Morris, 2016), or specific driver behaviour traits such as impulsiveness (Bıçaksız & Özkan, 2016), sensation seeking (Zhang et al., 2019), and aggressiveness (Alkinani, Khan & Arshad, 2020). Thus, their recommendations are limited to specific areas and cannot be generalized. Likewise, the few surveys that have covered several areas in the driver behaviour domain still need to provide in-depth analysis on reported experiments. This arises questions, such as: What sample sizes are previously used in the literature? What is the ratio of females to males in those presented samples? What are their age ranges and driving experiences? How many vehicles are used in those experiments? on which types of roads, these experiments are conducted? What is the time and duration of those experiments? Which driving data are collected in those experiments and which sensors are used for collecting these data? Answering such questions would certainly help future researchers in designing their experiments appropriately and in developing more efficient data acquisition systems (DAS). The current literature also lacks a survey that summarizes in-vehicle sensor-based driver behaviour articles, that consider the presence of various driver behaviour topics. The main motivation of this survey is a thorough examination of the publications, in which researchers have collected driving data using in-vehicle sensors, to provide a summary of its related topics, and to answer the aforementioned survey questions. The foremost objectives of this work are to suggest a classification that recognizes various aspects of this pertinent research area, summarize the common benefits, issues, and recommendations found in the literature; report researchers’ findings, present recommendations for researchers on how to plan their experiments adequately for data collection; finally, provide guidelines for future researchers interested in driver profiling and recognition.

The current survey is structured as follows. The introduction section provides the reader with a brief discussion about the topic, an explanation of existing gaps in the literature, states the motivation behind addressing these gaps, and outlines the objectives. The Survey Methodology section details the databases and the search query used for finding related articles, and also the criteria for filtering the resultant articles. The results of the proposed literature taxonomy are presented next to help researchers understand the main topics of this domain. The distribution of results shows the resultant statistics of the filtered articles. The Discussion section identifies the challenges faced by previous researchers, summarizes the benefits of conducting such researches, and outlines future recommendations for researchers. Significant scrutiny of the screened literature and recommendations on how to plan experiments appropriately is presented in the Substantial Analysis section. Then, future directions for researchers who are interested in driver profiling and recognition are outlined. Finally, the Conclusions section presents a summary of the conclusions formulated from this survey and the proposed future work.

## Survey methodology

Systematic review adopts methods to select, evaluate and synthesize ongoing experimental trials in the literature. It helps to answer research questions and presents an unbiased summary of findings through the use of inclusion/exclusion criteria.

### Information sources

The databases that are chosen for searching related articles, are IEEEXplore, ScienceDirect, Scopus, and Web of Science. These databases are selected due to their academic reliability and because they sufficiently cover the driver behaviour domain and offer a broad view of current literature.

### Search query

**Figure 1:**
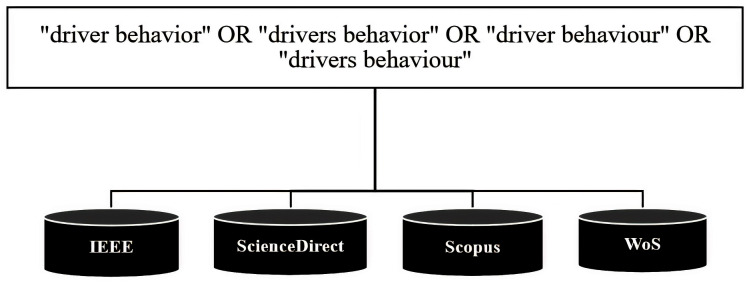
Search query.

Since the area of interest is driver behaviour, the search query is set to (“driver behavior” OR “drivers behavior” OR “driver behaviour” OR “drivers behaviour”). The reason for using the words “driver”, “drivers”, “behavior” and “behaviour” is because those words are used interchangeably in the literature. Fig. 1 shows the search query that is used in the selected databases. Table 1 shows the settings of the search query on the selected databases.

**Table 1:** Settings of search query.

Digital library	IEEE	ScienceDirect	Scopus	WoS
Search period	Jan 2014–Dec 2020	Jan 2014–Dec 2020	Jan 2014–Dec 2020	Jan 2014–Dec 2020
Language	English	English	English	English
Run on	Full text & metadata	Title, abstract & keywords	Title, abstract & keywords	Topic
Date of running search query	February 2021	February 2021	February 2021	February 2021

### Articles selection

The filtration process of articles consists of three repetitions of screening and filtering. Deletion of duplicated articles is performed during the first repetition. Unrelated articles are deleted in the second repetition after examining their titles and abstracts. The remaining filtered articles are then carefully read in the last repetition.

### Eligibility criteria for articles selection

A set of criteria is introduced to help decide which articles are to be included or excluded during the three repetitions of screening and filtrating.

- Eligibility criteria on research domain: articles are excluded if they are in eco-driving, vehicle to vehicle, autonomous driving, or augmented reality domains.
- Eligibility criteria on data sources: only experimental studies are included, thus excluding articles in which driving data are obtained from predesigned questionnaires, observations, simulations, or data from existing NDS.
- Eligibility criteria on sensors used: articles that have not identified driver behaviour based on vehicular sensors are excluded, such as articles that rely on wearables, eye tracking, or smartphone sensors.
- Eligibility criteria on vehicles’ type: articles on which experiments are done specifically on electric vehicles, or heavy vehicles such as buses, are excluded.
- Eligibility criteria on articles’ language: only English-written articles are included.

Table 2 shows detailed inclusion/exclusion criteria.

**Table 2:** Inclusion/exclusion criteria.

Inclusion criteria	Exclusion criteria
Only English written articles	Non-English written articles
From January 2014 to December 2020	Articles before 2014 and after 2020
IEEEXplore papers were journals and magazines. ScienceDirect papers were research articles. Scopus papers were articles. Web of Science papers were articles.	Eco driving, vehicle to vehicle, autonomous driving or augmented reality domains. Predesigned questionnaires, observations, simulations or data from existing NDS. Head movement, wearables, gloves, heart rate, eye tracking, EEG/ECG or smartphone sensors. Electric vehicles or heavy vehicles.

### Search results

**Figure 2:**
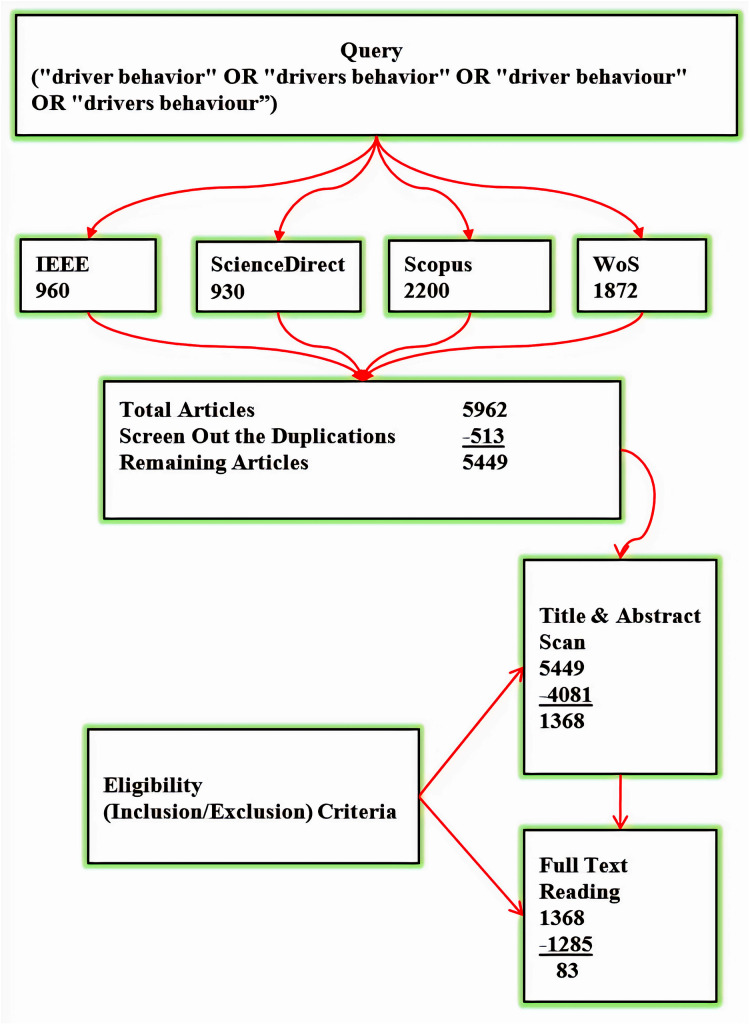
Search results.

The result of applying the search query is 5,962 articles from the four selected databases in the first repetition. The remaining articles after removing the duplicates in the first repetition are 5,449. The result of scanning articles’ titles and abstracts in the second repetition is the exclusion of 4,081 articles. Further 1,285 articles are excluded following full-text reading on the third repetition. The remaining articles are 83. Figure 2 illustrated the entire process of search results.

## Results of literature taxonomy

**Figure 3:**
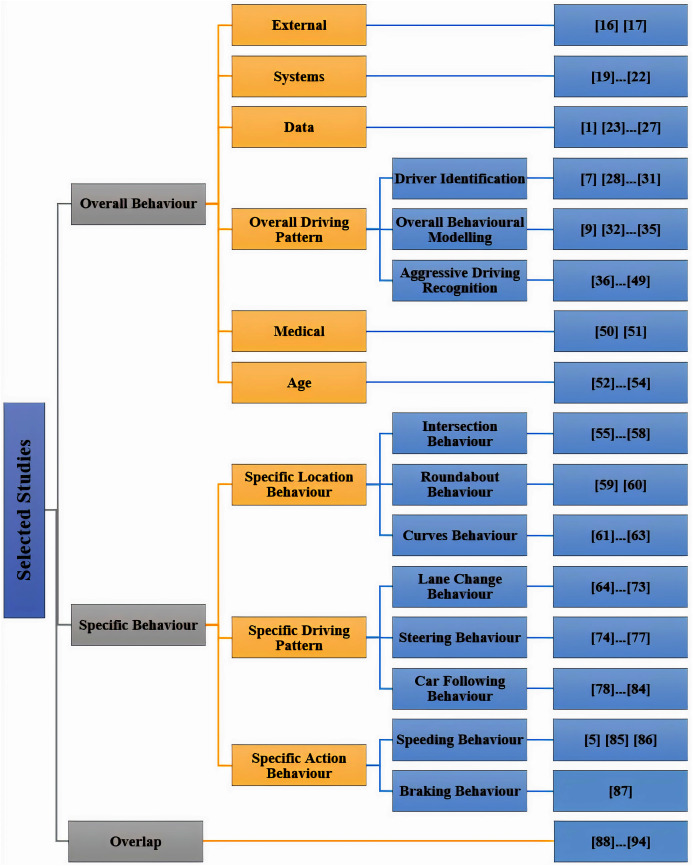
Taxonomy of final articles.

A taxonomy is constructed and presented based on the authors’ perspective of the 83 articles. This is to achieve one of the objectives of this survey, which is a classification for in-vehicle sensor-based driver behaviour publications, in order to help future researchers understand the various topics of this domain. The taxonomy that is proposed in Fig. 3, is constructed based on the articles’ main topic. The presented taxonomy confirms three main categories, the first category is named ‘overall behaviour’, which contains articles that aim to study the overall behaviour of drivers instead of focusing on one particular behavioural aspect. The second category is named ‘specific behaviour’, which contains articles that are directed to the study of specific behavioural aspects instead of the overall behaviour of drivers. The third and final category is named ‘overlap’ which contains articles that traverse the two previous categories.

### Overall behaviour

The first main category in the taxonomy is related to articles that study the overall behaviour of drivers instead of focusing on one particular behavioural aspect. As expected, such studies have different goals which is why this category has six subcategories, namely, external, systems, data, overall driving pattern, medical, and age. The number of articles in this category is (*n* = 42/83).

#### External

External subcategory (*n* = 2/42) is related to studies that measure the impact of engaging in a secondary task on driver behavior, such as using a cellphone or adjusting radio channels. A portable system for controlling and monitoring a driver’s cellphone is proposed in Khandakar et al. (2019), the system gathers data and sends it to a mobile phone to detect driver behaviour. In Bastos et al. (2020), driving data were collected to investigate cellphone use in Brazil and results are compared with those reported in international studies.

#### Systems

Systems subcategory (*n* = 5/42) is related to studies that measure the impact and benefits of vehicle assistance systems on driver behaviour. Researchers compare the impact of adaptive cruise control (ACC) system on driver behaviour and traffic flow when the ACC is on and off in Schakel et al. (2017). Similarly, Varotto et al. (2017) identify the main factors that influence driver’s choice in activating and deactivating the ACC system. A comprehensive modeling framework is proposed in Varotto et al. (2018) which describes the underlying decision-making process of drivers, with full range ACC at the operational level, based on risk allostasis theory (RAT). Galarza & Paradells (2018) propose and implement a system that reduces the amount of information offered to drivers when interacting with in-vehicle information systems (IVIS). Improvements on visual warning systems are proposed in Tonguç, Akçay & Gürbüz (2018), to identify adverse driving conditions and warn other drivers moving on the same route.

#### Data

Data subcategory (*n* = 6/42) is related to studies that aim to store and process collected driving data. Two low-cost DASs that fused driving data, for driver behaviour analysis, are proposed in Carmona et al. (2015) and Andria et al. (2016); The first gathered data from on-board diagnostics (OBDII) and inertial measurement unit is (IMU) (Carmona et al., 2015), while the latter gathered data is from controller area network (CAN) bus, IMU and GPS (Andria et al., 2016). To help reduce synchronization errors of sensors’ data, an automated method that relies on offline and online synchronization of data streams is proposed in Fridman et al. (2016). Moreover, Xiao et al. (2020) propose a data fusion-based approach, which integrated GPS data with OBDII readings, to build a model that adapts to data fluctuations and errors in real-time experiments. Kim & Baek (2020) also propose a system that automatically extracts data from sensor-based vehicles for behavioural analysis. Furthermore, an unsupervised method for segmenting naturalistic driving data, that targeted the formation of high-level understanding of driver behaviour, from low-level sensor data, is introduced in Bender et al. (2015).

#### Overall driving pattern

This subcategory (*n* = 24/42) describes articles related to identifying, analyzing, and modeling driving behavioural patterns. Articles in this subcategory are further classified into three subgroups, namely, driver identification, overall behavioural modeling, and aggressive driving recognition.

The first subgroup, driver identification, is related to studies that analyzed driving data to identify an individual’s driving pattern. The first three articles (Cai et al., 2018; Li et al., 2018; Büyükyildiz et al., 2017) propose methods to identify drivers based on their driving data, *i.e*. personal ‘fingerprint’; the first two studies, Cai et al. (2018) and Li et al. (2018) use machine learning methods, while the third study (Büyükyildiz et al., 2017) use statistical methods to identify, and draw conclusions on, various driving patterns. Similarly, Liu et al. (2017) proposed a visualization method, called deep sparse autoencoder (DSAE), to recognize of distinctive driving behavioural patterns in continuous driving data. Moreover, a graphical modeling method that can illustrate individuals’ driving habits by extracting and ranking their typical driving patterns is proposed in Chen et al. (2019).

The second subgroup, overall behavioural modeling, is related to studies that analyzed driving data for modeling various behavioural patterns. Park et al. (2016) modeled behavioural patterns during unexpected traffic emergencies by analyzing drivers’ pedal behaviour in remodeled test vehicles. An improved unsupervised deep learning model that analyzed interactions between road environment and driver behaviour throughout the generation of graphical representations is proposed in Bichicchi et al. (2020). Moreover, an unsupervised learning method, that presumes a two-layered hierarchical structure, called a double articulation analyzer with a temporal prediction (DAA-TP), analyzed driving data and predicted how drivers behaviour changes on a temporal axis, is proposed in Taniguchi et al. (2014). Also, Wu & Jovanis (2016) proposed a flexible cohort-based analysis structure, that analyzes and models behavioural patterns in NDS and announces warnings through the onboard safety warning systems. Furthermore, Bella & Nobili (2020) model drivers’ behaviour with relation to a pedestrian crossing, a designated zebra crossings.

The third subgroup, aggressive driving recognition, is related to studies that analyze driver behaviour to identify and differentiate aggressive driving patterns from normal driving patterns. Zylius (2017) proposed a machine learning approach to identify the aggressive and safe driving pattern by using features extracted from inertial signals (3-axis acceleration sensor); two levels of driving patterns are proposed (safe driving, aggressive driving). A driving behaviour-based event data recorder (DBEDR), which recognizes seven different driving behaviours and a danger level, is proposed in Wu, Chen & Yeh (2013). Similarly, Gündüz et al. (2017) present a risk assessment model that differentiates various driving patterns. The model categorizes aggressiveness into three groups (low risk, medium risk, and high risk), the study relies on legal authority reports that identified risks based on possible collision damages. Also, a clustering-based grading method that evaluated driving performances of novice drivers against an experienced driver baseline, producing a GPA on a 0–4 scale, is proposed in Liu & Hansen (2019). Moreover, Zheng et al. (2017) assessed how driver type and driver behaviour are related in an environment of high levels of pedestrian-vehicle interactions; this is done *via* the use of a 1 to 10 scale; with 10 corresponding to the most aggressive style and 1 corresponding to the least aggressive style. Furthermore, four major risky driving behaviours, are given a score of 1 to 10, and their associated risk levels are identified and evaluated in Yuksel & Atmaca (2020); a fuzzy logic-based risk assessment model is established based on the study findings. Silva & Eugenio Naranjo (2020) introduced a data-driven machine learning methodology for classifying driving patterns into three types (calm, normal, and aggressive). Also, Lee & Jang (2017) developed a framework for identifying potentially aggressive driving behaviours and provided drivers with feedbacks that guide them towards adopting less aggressive driving; drivers are categorized into three levels of aggressive behaviours (high, medium, low). Moreover, Li et al. (2015) allowed risk consulting experts to assign risk scores to participate drivers (from 1 to 5, where 1 being least aggressive and 5 being the most aggressive). Furthermore, González et al. (2014) model the effect of aggressiveness on driving parameters, such as speed, longitudinal and sideways accelerations, which cause a scaling transformation on their recorded files. A methodology to track acceptable and non-acceptable driving behaviour and calculate risk model using the envelope of the data and nota prior thresholds, is proposed in Joubert, Beer & Koker (2016). Zardosht, Beauchemin & Bauer (2018) proposed a method that analyzes statistical features of CAN signals to explore two distinct driving patters (aggressive and moderate). An algorithm that evaluates the driver aggressiveness using scores, called handling risk factors (HRF), to characterize driver behaviour is presented in Hong et al. (2016a); high HRF values imply aggressive driving pattern, while low HRF values imply safe driving pattern. Finally, a method that adapts dynamic time wrapping (DTW) and hidden Markov model (HMM) to classify drivers into five different behavioural patterns (timid, cautious, aggressive, best, assertive) is proposed in Yao et al. (2020).

#### Medical

Both studies in the medical subcategory (*n* = 2/42) are related to the effects of certain diseases on driver behaviour. Multiple sclerosis (MS) is a potential disease that disables the brain and spinal cord, and as such, Krasniuk et al. (2019) undertook an on-road assessment that incorporated strategic driving maneuvers to understand the underlying differences between MS drivers who passed the driving maneuvers against those who failed them. Sleep apnea is a serious sleep disorder, and McLaurin et al. (2018) used topic modeling to identify the effect of sleep apnea on driving, by comparing trips of drivers who suffer from obstructive sleep apnea against drivers who do not have it.

#### Age

Age subcategory (*n* = 3/42) is related to studies that aim at understating the effect of age on driver behaviour. Svetina (2016) displayed how driver’s behaviour tends to progress over the years, by comparing the reaction times of drivers of different age groups in experimental settings. Also, Merickel et al. (2019) assessed driving patterns in people with age-related dysfunction, by analyzing vehicle control data such as steering, braking, and accelerating in older adults with a range of cognitive and visual functional abilities. Moreover, Farah et al. (2014) evaluated the effect of parents guiding teens to adopt vigilant care in driving, especially during their first year of driving.

### Specific behaviour

The second main category contains articles (*n* = 34/83) that study specific behavioural aspects instead of the overall behaviour of drivers. This category has three subcategories based on their goals, namely, specific location behaviour, specific driving pattern, and specific action behaviour.

#### Specific location behaviour

This subcategory (*n* = 9/34) is related to studies that focus on analyzing driver behaviour solely on specific geometric locations. Articles in this subcategory are further classified into three subgroups namely, intersection behaviour, roundabout behaviour, and curves behaviour.

The first subgroup, intersection behaviour, contains articles that analyze driver behaviour solely at intersections. A hybrid state estimation (HSS)+HMM framework is proposed to estimate driver behaviour near intersections (Gadepally, Krishnamurthy & Ozguner, 2013). A methodology for analyzing driver behaviour by distance gaps accepted by the majority of participating drivers, at a signal-based intersection and two-way stop-controlled intersections, is proposed in Dabbour (2015). Moreover, a system dedicated to predicting driver intention at non-signalized t-intersections, by integrating clustering and classification models, is proposed in Yi et al. (2018). Furthermore, a method for estimating driver behaviour with relation to pedestrians crossing intersections is proposed in Lubbe & Rosén (2014).

The second subgroup, roundabout behaviour, contains articles that aim to analyze driver behaviour solely at roundabout locations. Statistical analysis and evaluation of driver’s behaviour when crossing roundabouts is presented in detail in Silva et al. (2014). Also, Zyner, Worrall & Nebot (2019) presented a method for predicting driver intention at roundabouts through the use of recurrent neural networks combined with a mixture density network output function.

The third subgroup, curves behaviour, contains articles that aim to analyze driver behaviour solely at geometric curves. Li et al. (2019) developed a data-driven trajectory model based on long short-term memory neural network (LSTM NN) to study the path characteristics for experienced drivers when driving on curved two-lane roads. Moreover, two driver behaviour models are proposed in Cerni & Bassani (2017) to investigate the causes of differences between vehicle trajectories and road alignments and to estimate vehicle trajectories on curve roads. Furthermore, Montella et al. (2014) developed a model for predicting drivers’ speed on curves, by using deceleration and acceleration rates in the operating speed profiles.

#### Specific driving pattern

This subcategory (*n* = 21/34) describe articles that are related to analyzing specific behavioural pattern. The articles in this subcategory are organized into three subgroups namely, lane change behaviour, steering behaviour, and car-following behaviour.

The first subgroup, lane change behaviour, contains articles that aim to analyze drivers’ lane changing behaviour. Satzoda & Trivedi (2014) proposed a method that extracts data from sensors, to analyse risky lane change behaviour from highway drives. A fusion approach that utilizes data, from a camera and an OBDII, which detects drivers’ lane changing behaviour using a collaborative representation classifier (CRC) is proposed in Gao, Murphey & Zhu (2018). Also, driving behaviour awareness (DBA) model, on a dynamic Bayesian network and distributed GA, to estimate driver’s behaviour in lane changing scenarios is proposed in Xie et al. (2018). Moreover, Hill, Elefteriadou & Kondyli (2014) measured distributions of duration and gap acceptance in lane-changing scenarios and examine the characteristics of conservative and aggressive drivers, according to their lane-changing behaviour. Zhao et al. (2016) and Yao et al. (2016) studied lane changing behaviour at the tactical level with on-road perspective. The first publication (Zhao et al., 2016) addresses vehicle trajectory collection, while the second one (Yao et al., 2016) proposes an automatic method for extracting lane change segments from continuous driving. A fusion approach that utilizes multiple differing modality data for detecting driver’s lane change behaviour *via* the use of a dimensionality reduction model, is proposed in Gao, Murphey & Zhu (2019). Moreover, Wang et al. (2019) proposed a theoretical lane change warning model, that determines warning thresholds for dangerous lane changing behaviours. Also, researchers have examined the effect of integrated collision, on teenage drivers, in lane-changing maneuvers, in Jermakian et al. (2017). Finally, researchers compute left lane occupation time, by performing calibration of overtaking acceleration models, in Llorca, Moreno & Garcia (2016).

The second subgroup, steering behaviour, contains articles that aim to analyse drivers’ steering behaviour. Katzourakis et al. (2013) proposed a method to quantify driver’s arm neuromuscular admittance during steering in real-time driving. A deep learning-based method to identify driver’s steering behaviour during near rear-end collision maneuvers is proposed in Hassan et al. (2017). As well, the time-dependent concept of driver decoupling/recoupling using a steer-by-wire system combined with override recognition by counter steering above a certain threshold is proposed in Heesen et al. (2014). Moreover, a method to aid drivers in handling critical understeering situations is developed and verified in Hildebrandt et al. (2015).

The third subgroup, car-following behaviour, contains articles that analyze and model drivers’ car-following behaviour. A driver-vehicle diagnostics system for car-following behaviour based on Gaussian mixed model (GMM) and probability distribution functions of dynamic characteristics is proposed in Butakov & Ioannou (2014). Bifulco et al. (2014) presented an NDS, focusing on car-following behaviour for single vehicles on long road segments, to obtain microscopic data to be used in statistical analysis and calibration of driving models. Similarly, through a genetic algorithm calibration procedure, experimental data are collected and used to calibrate the parameters of the prospect theory car-following model, in Schorr, Hamdar & Silverstein (2014). Also, Errampalli, Mallela & Chandra (2020) developed a modified car-following model for heterogeneous traffic conditions in India, based on the general motors (GM) model and Hidas model, using data collected on urban and non-urban corridors. Moreover, a coupla-based methodological framework is presented in Das et al. (2019) for the safety evaluation of car-following behaviour in non-lane-based traffic. Furthermore, Qi et al. (2015) identified common and individual driving patterns (moderate, cautious, and aggressive patterns) between drivers in car-following scenarios by developing an ensemble clustering method (ECM) for data mining; based on fuzzy c-mean algorithm (KFCM) and modified latent dirichlet allocation (LDA). Finally, an analysis of the impact of roadway characteristics on drivers’ car following behaviour is presented in Wang et al. (2014).

#### Specific action behaviour

This subcategory (*n* = 4/34) is related to studies that focused on analyzing a specific driving action. Articles in this subcategory are further classified into two subgroups namely, speeding behaviour and braking behaviour.

The first subgroup, speeding behaviour, contains articles that focus solely on speeding behavior analysis. A two-level strategy, that predicts the risk of overspeeding before the occurrence of accidents, using the dempster-shafer theory (DST) and the fuzzy theory (FT), is presented in Derbel (2018). Moreover, Stigson et al. (2014) apply not speeding as inceptive for pay as you speed (PAYS) concepts. Furthermore, Stephens et al. (2015) examined the effect of anxiety, mood, and anger on speeding behaviour.

The second subgroup, braking behaviour, contains an article that focuses solely on analyzing braking behaviour. Peng et al. (2019) propose a rough set-based method for collision risk assessment by inferring braking action in near-crash situations.

### Overlap

The third and final category of the taxonomy (*n* = 7/83) contains articles that traverse the two previous main categories or their subcategories. The study that measured stopping behaviour at intersections is Hong et al. (2016b), which measures how sensitive elder drivers are, in their approaching and stopping behaviour at intersections, when compared with younger drivers. Two studies provide initial results on the effect of speeding behaviour on different types of geometrical curves (Turner, Woolley & Cairney, 2015; Maljković & Cvitanić, 2016). One study uses the results of a small-scale NDS to evaluate the performance of existing models on two specific behaviours, car-following behaviour and curve behaviour (Fleming et al., 2019). A novel collision warning algorithm that employs a new index, named RDSI, to evaluate driving risk levels on two specific behaviours, car-following and lane changing, is proposed in Wang et al. (2016). Lyu et al. (2019) have explored the effect of advanced driver assistance systems (ADAS) on braking behaviour and driving performance in lane-changing scenarios. The last article in this category (Salmon et al., 2017) examines the impact of providing concurrent verbal protocols on driving performance, on two different locations, *i.e*. signalized intersections and roundabouts.

## Distribution of results

**Figure 4:**
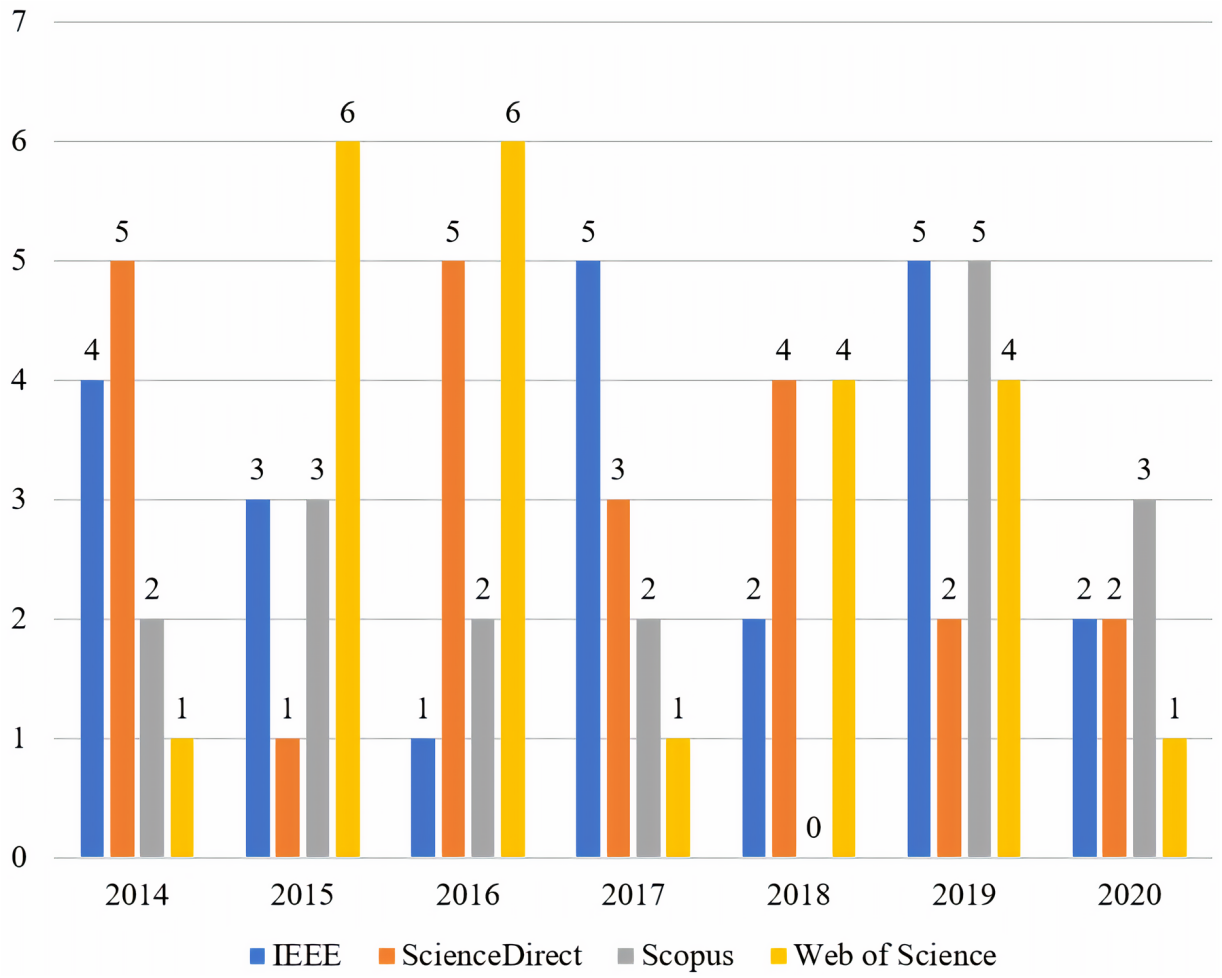
Distribution of the finalized articles across the main categories of the proposed taxonomy.

**Figure 5:**
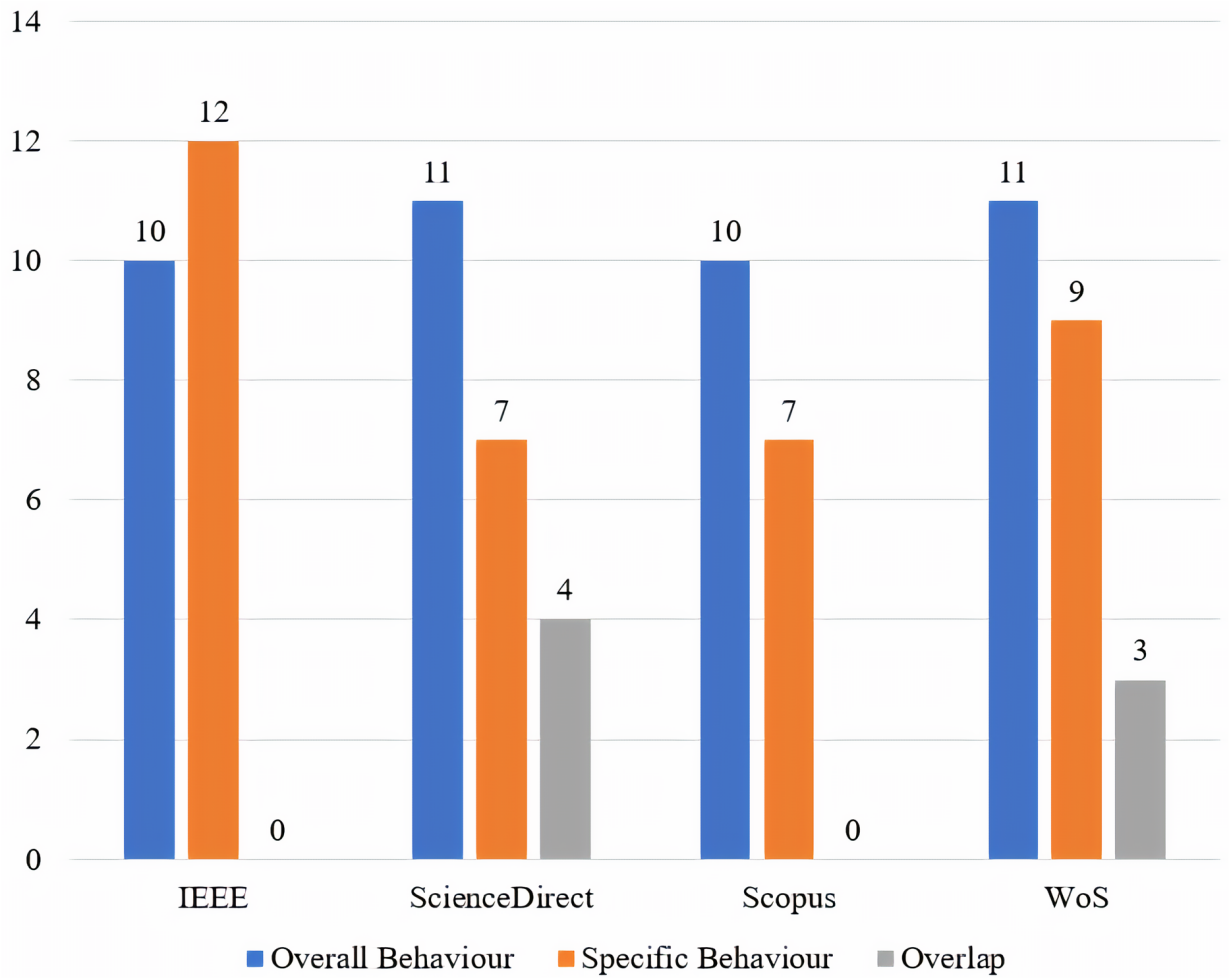
Distribution of the finalized articles from 2014 to 2020.

The distribution of the finalized articles, across the main categories of the proposed taxonomy, is presented in Fig. 4. The distribution of the finalized articles, in the four selected databases, from 2014 until 2020 is presented in Fig. 5.

## Discussions

This section discusses the common motivations, challenges, and recommendations in the reported literature because they are particularly valuable for future studies. The motivations section informs imminent researchers on why the researchers before them committed themselves to this field of science and what inspired them to follow their treasured research in this area. The challenges section helps future researchers in identifying the common issues they could face during their research journey. Since previous researchers admit their limitations yet define critical suggestions, the recommendations section serves as a guide that helps resolve certain issues. It represents the association connecting the new researchers with previously involved ones. This section also contains an additional part that compares various survey articles, related to the driver behaviour domain in existing literature, to the current survey.

### Motivations

Investigating drivers’ behaviour have numerous benefits. This section lists some of the important benefits that encouraged researchers toward this domain. They are grouped into four subsections. The corresponding references are cited for further discussions.

#### Benefits of improving traffic safety

Road traffic injuries remain the leading cause of death for young people between the age range of 17 to 29. Furthermore, drivers over the age of 76 have the highest fatal crash rates per mile of driving. A major factor that contributes to crashes is distractions, and it is becoming paramount; at this point, to analyze the effects of distractions on driving performance, especially in scenarios where drivers are talking while driving, such studies can provide insight on how to limit the use of secondary tasks while driving (Khandakar et al., 2019). Researchers need to pursue this domain, to better understand the human factors that contribute to crashes, with the hopes of reducing future traffic accidents and improving pedestrians’ and passengers’ well-being, because drivers’ behaviour has a strong impact on traffic safety (Silva & Eugenio Naranjo, 2020). Moreover, analyzing specific behaviours, such as car-following, or analyzing drivers’ behaviour concerning specific geometric locations, such as intersections, would help understand how certain behaviours contribute more than others to crashes, and why crashes occur more often on particular locations Gadepally, Krishnamurthy & Ozguner (2013) and Silva et al. (2014). Also, identifying different driving styles and patterns would help make tailored policies and regulations for drivers of certain ages and driving experiences, and guidelines for correcting dangerous behaviours. Researchers should also study the impact of geometric roadway design on driver behaviour, which would help build more suitable road constructions for safe driving (Cerni & Bassani, 2017).

#### Benefits of managing data

During experiments, sensors collect raw data. The more experiments the researchers perform, the more data filtration must be done, which is time-consuming and costly. Developing a DAS that fuses sensors efficiently together and decreases raw data overhead, is vital in NDS. Therefore, researchers should aim to improve data management processes such as data extraction, sensors’ fusion, and synchronization (Fridman et al., 2016).

#### Benefits related to enhancing assistance and navigation systems

Vehicles nowadays are packed with various assistance/navigation systems, such as ACC, collision avoidance systems (CAS), and ADAS. These systems assist drivers in parking and driving. Because such systems interact with drivers, it is possible to study the effects of existing assistance systems on their behaviour, such as, understanding the effect of ACC activation/ deactivation on traffic congestion and measuring the influence of ACC on the efficiency of traffic flow (Schakel et al., 2017). Furthermore, researchers can introduce enhancements for the current assistance systems, such as developing a prediction system that is dedicated to measuring drivers’ intention at specific locations, such as non-signalized T-intersections (Yi et al., 2018) or determining the effects of integrated collision warning systems on teenage drivers (Jermakian et al., 2017). Researchers can also measure the impact of introducing new assistance systems on drivers’ behaviour, such as measuring the impact of using steer-by-wire systems as a concept for driver de-/recoupling in automatic evasive maneuvers (Heesen et al., 2014). Furthermore, researchers can propose a novel algorithm, that employs a new index in its evaluation of risky driving, in collision warning systems (Wang et al., 2016). Researchers should also aim to develop a conceptual model that clarifies driver decisions when interacting with collision and control systems.

#### Benefits related to the health sector

Some diseases have an adverse effect on patients’ cognitive and neuromuscular functions which would inevitably affect their driving performance. The study of the effect of certain diseases on driving is of prime importance for advancing the medical sector and the safety of patients. For instance, MS attacks the protective sheath (myelin) covering the nerve fibers, thus, causing problems in communication between the brain and the remaining parts of the body. Researchers compare MS patients who succeeded *versus* those who failed on-road assessment in Krasniuk et al. (2019). Similarly, sleep apnea, affects patients’ alertness and performance, so researchers have used topic modeling to identify patterns from drivers with OSA during driving trips (McLaurin et al., 2018). More studies on how other diseases affect driving behaviour should be investigated as well to provide more insight for medical practitioners on how to find better treatments for their patients.

### Challenges

Researchers face numerous challenges when investigating this domain. This section presents some of the up-to-date issues, which are grouped into four subsections, along with citations for further discussions.

#### Issues relevant to modeling driver behaviour

Relying on official records can provide very limited insight on drivers’ behaviour during accidents, for example, police reports rarely provide data on when drivers are distracted before accidents, and the occurrences of such distractions (Bastos et al., 2020). One of the reasons why driver behaviour results vary; from one study to another, is the difficulty of distinguishing different driving styles and anticipating other drivers’ behaviour on the road. This can lead to invalid assumptions, contradictory findings, and data that cannot be used globally. Another issue is the use of small sample sizes which can also lead to similar issues (Farah et al., 2014). Moreover, it is at times difficult to deliberately induce critical or unsafe behaviour in road experiments, due to ethical reasons, resulting in experiments being simulated and conclusions being limited or biased (Bifulco et al., 2014).

#### Issues related to assistance and navigation systems

There is a need to develop more reliable and fast responding assistance systems. ADAS development is not straightforward and has many issues. They are limited by user’s acceptance due to their unreliability, such as detection mistakes occurring in bad weather conditions, which lane-keeping systems are usually prone to (Khandakar et al., 2019). Moreover, ACC systems have an opposing effect on traffic flow efficiency (Schakel et al., 2017). Also, most ADAS are preconfigured to be used by average drivers while dismissing the individual characteristics of particular drivers, *i.e*. no personalization features because they use simplistic algorithms (Wang et al., 2016). Furthermore, forward collision warning (FCW) systems suffer from a high rate of false-positive alarms (Lyu et al., 2019). Such issues are major factors in limiting ADAS acceptance in the driving community. Future researchers need to address these issues to improve drivers’ trust in those systems.

#### Issues of existing systems

There are very few systems available on the market which claim to monitor driver behaviour. Most of these systems use a simple sensor to monitor the drivers’ behaviour, such as a GPS. They are mostly used for tracking drivers in fleet management. Those systems are not effective in preventing accidents, and they are used for monitoring purposes only. Currently, there are no sophisticated commercial systems, that utilize multiple sensors, such as lidars, accelerometers, and OBDII, to reliably collect data and measure driving behaviour. However, researchers that have introduced DAS in their studies, reported complications in using some of the sensors. Those issues are reported in the section “issues related to data acquisition and handling”. Other vehicular systems, such as ADAS and FCW have issues of their own and can impact negatively on drivers’ behaviour, those challenges are reported in section “issues related to assistance and navigation systems”. One of the areas that future researchers can work on, is building a reliable driver behaviour evaluation system that consists of two subsystems. The first subsystem is a DAS that utilizes multiple sensors to collects driving data. The sensors of the DAS subsystem should be able to operate on most vehicle brands, thus making the system platform-independent. The second subsystem is an AI-based recognition system that analyzes the collected data to evaluate drivers’ performances and help prevent accidents. Manufacturers should aim to develop such a system and make it available on the market.

#### Issues related to data acquisition and handling

There are several methods to acquire data, such simulations, questionnaires, and experiments. Here are the issues researchers need to consider when choosing a method for data collection. The use of simulators, throughout the literature, is mentioned as a limitation (Katzourakis et al., 2013). Shortcomings in simulation use, include physical limitations and realism, simulator sickness, and data accuracy. Questionnaires provide subjective and biased judgments of drivers (Qi et al., 2015). It is preferable to collect data in real-time experiments. However, there are challenges that researchers need to consider when deciding to collect data in a naturalistic manner. For example, a smartphone’s accelerometer requires high-performance computational capability (Khandakar et al., 2019). Physiological/biomedical sensors add complexity because of the additional instrumentation required and their effect on driver’s comfort and performance (Galarza & Paradells, 2018). There are also other factors that researchers need to consider when selecting the sensors for their proposed DAS, such as transmission overhead, complexity, reliability, and cost. GPS poses an unaffordable burden for transmission overhead and data storage (Xiao et al., 2020). Smartphone sensors have overestimation issues when compared with fixed devices (Joubert, Beer & Koker, 2016). Researchers also need to think about the synchronization of incoming data streams and the exclusion of irrelevant data that sensors usually record. Such issues make the analysis of experimental data time-consuming and costly (Satzoda & Trivedi, 2014). Moreover, it is challenging to use computer vision techniques while maintaining accurate and robust results because the line of sight must be aligned with the camera’s field of view and cannot be blocked (Gao, Murphey & Zhu, 2018). Furthermore, there are few distance sensors, such as 3D lidars, that are considered very costly (Zhao et al., 2016). Another issue is that some experiments require the presence of the researcher in the vehicle along with the driver, which could influence the results (Schorr, Hamdar & Silverstein, 2014). Therefore, researchers should carefully plan and select the sensors of their proposed DAS with the aforementioned factors in mind.

### Recommendations

This section presents a summary of the important recommendations that mitigates current challenges, grouped into three subsections based on the party they are addressed to.

#### Recommendations to researchers

With regards to data collection, it is recommended to:

- Develop a general model which determines the amount of data required in experimental studies (Schakel et al., 2017).
- Use large sample sizes, in experiments, for more reliable results (Galarza & Paradells, 2018).
- Include more sensors in proposed DAS to increase data collection accuracy (Li et al., 2018).
- Include a wider range of drivers to ensure the sample is diverse in age and gender representation (Büyükyildiz et al., 2017).
- Adapt blockchain technology to securely store driver history and data (Yuksel & Atmaca, 2020).

With regards to driver behaviour modeling, it is recommended to:

- Combine driving signals with psychological models (Li et al., 2015).
- Add more parameters when analyzing data for information, and more features when proposing classification models, and more metrics when evaluating these proposed models (González et al., 2014; Zardosht, Beauchemin & Bauer, 2018; Krasniuk et al., 2019).
- Investigate whether aging has a significant effect on driving performance (Svetina, 2016).
- Improve lane-change detection accuracy in current models while fully capturing the lane change decision-making process (Hill, Elefteriadou & Kondyli, 2014).

With regards to existing research, previous researchers have either recommended overcoming current research limitations or extending the current research

- Researchers have suggested future researchers overcome some of the limitations in their research, such as overcoming the concern of how aggregating driving data, over several driving trips, would mask the effect of individual trip data (Wu & Jovanis, 2016), overcoming the assumption of clear driver decision in the estimation process of driver decision in near intersection scenario (Gadepally, Krishnamurthy & Ozguner, 2013) and overcoming the issue that humans may change their neuromuscular behaviour during the estimation window of driver’s arm admittance (Katzourakis et al., 2013).
- Researchers have suggested extending their experimental research to other countries using different types of vehicles and experimental setups (Schorr, Hamdar & Silverstein, 2014).
- Also, researchers have oftentimes encouraged future researchers to undertake research, similar to their own, intending to validate their findings and confirm their conclusions Wang et al. (2016) and Salmon et al. (2017).

#### Recommendations to developers

Car manufacturers should aim to develop an ADAS, with personalization features, that reliably evaluate drivers’ performance. Developers need to understand the reasons why drivers choose to activate or deactivate assistance/navigation systems, such as ACC (Varotto et al., 2017), in certain scenarios, and how to encourage them to rely more often on these systems. They should also aim to address the issues behind ADAS low acceptance in the driving community (Lyu et al., 2019), such as false alarms and inaccurate lane detection.

#### Recommendations to safety and policy makers

More training courses should be implemented by policy makers before issuing licenses to drivers, especially adolescences, such as, guidelines on how to keep minimum safe distances between vehicles at intersections, and educational programs that increase the overall awareness of the importance of safe driving and the importance of following traffic rules and regulations (Galarza & Paradells, 2018).

### Survey analysis

In this section, various survey articles, related to the driver behaviour domain in existing literature, are compared to the current survey. Existing surveys mostly focus on reviewing specific topics in the driver behaviour domain, rather than the entire domain, such as investigating drivers’ anger (Demir, Demir & Özkan, 2016). Some articles review specific behaviours, such as car-following behaviour (Saifuzzaman & Zheng, 2014), lane changing behaviour (Koesdwiady et al., 2016), and intersection behaviour (Shirazi & Morris, 2016), while others review specific driver traits such as impulsiveness (Bıçaksız & Özkan, 2016), sensation seeking (Zhang et al., 2019), and aggressive driving behaviour (Alkinani, Khan & Arshad, 2020). Few articles review the use of machine learning-based technology in ITS Nguyen et al. (2018), Martinez et al. (2017), Alsrehin, Klaib & Magableh (2019) and Pamuła (2016), the evolution of vehicles’ sensing technology and their effect on safety (Massaro et al., 2016), and collision avoidance in assistance systems for intelligent vehicles Dahl et al. (2018), Zhao et al. (2017) and Mukhtar, Xia & Tang (2015). The effect of policies on driver behaviour and safety has been reviewed in Shinar & Gurion (2019). The effect of roadside vegetation, roadside advertisements, and road markings on safety have been reviewed in Van Treese et al. (2017), Oviedo-Trespalacios et al. (2019) and Babić et al. (2020) respectively. Furthermore, one article reviews the importance of big data and its analysis in the driver behaviour domain (Terzi, Sagiroglu & Demirezen, 2018). It is important to note, only a few survey articles aim to cover various topics in this domain, such as examining various driving patterns to identify links between driving styles and road safety (Sagberg et al., 2015), examining driver behaviour in mixed traffic conditions Munigety & Mathew (2016) and Asaithambi, Kanagaraj & Toledo (2016), looking into trends and developments in road user behaviour and traffic safety (Van Haperen et al., 2019) and systematically reviewing articles based on knowledge graph to identify trends in driver behaviour domain (Liu et al., 2020). However, this complete survey is prepared to provide material on the major driver behaviour research topics that are covered in part or not covered in the above surveys. This present survey covers most topics related to driver behaviour domain and suggests new directions for research and development in the future. It creates a new classification system for recently completed works on the basis of data analysis obtained from various studies. It also embodies what the present literature lacks, which is a substantial analysis that provides guidelines for researchers on how to set their experiments appropriately. Moreover, it explores the current trends’ challenges, motivations of those trends and suggests recommendations for researchers. Furthermore, it proposes future research directions for driver profiling and recognition.

The main contributions of the current survey are:

1. Provides a coherent taxonomy that classifies existing literature with regards to sensor-based driver behaviour domain into several categories and subcategories based on related topics.
2. Identifies the common challenges in current trends, explores the motivations in using those trends, and summarizes recommendations for future trends.
3. Provides a substantial analysis section that provides answers to gaps found in the current literature.
4. Offers future directions for researchers interested in driver profiling and recognition.

## Substantial analysis

**Figure 6:**
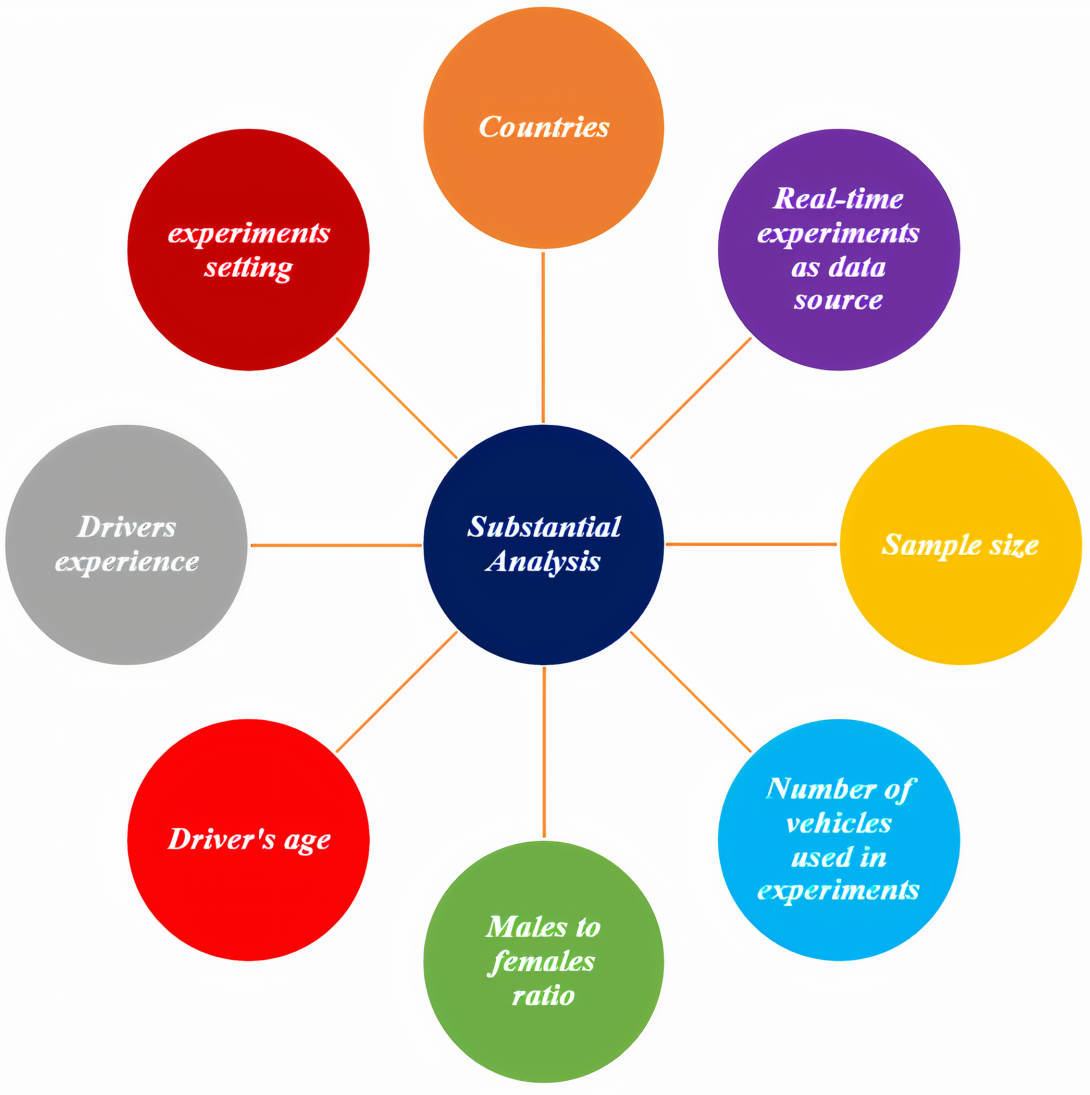
The eight parts of the substantial analysis.

A substantial analysis is presented to answer the survey questions, presented in the introduction section. This section provides guidelines that would help researchers design their experiments and provides suggestions for further future work. This analysis is categorized into eight parts to provide detailed information on the reported experiments, as shown in Fig. 6.

### Countries

**Figure 7:**
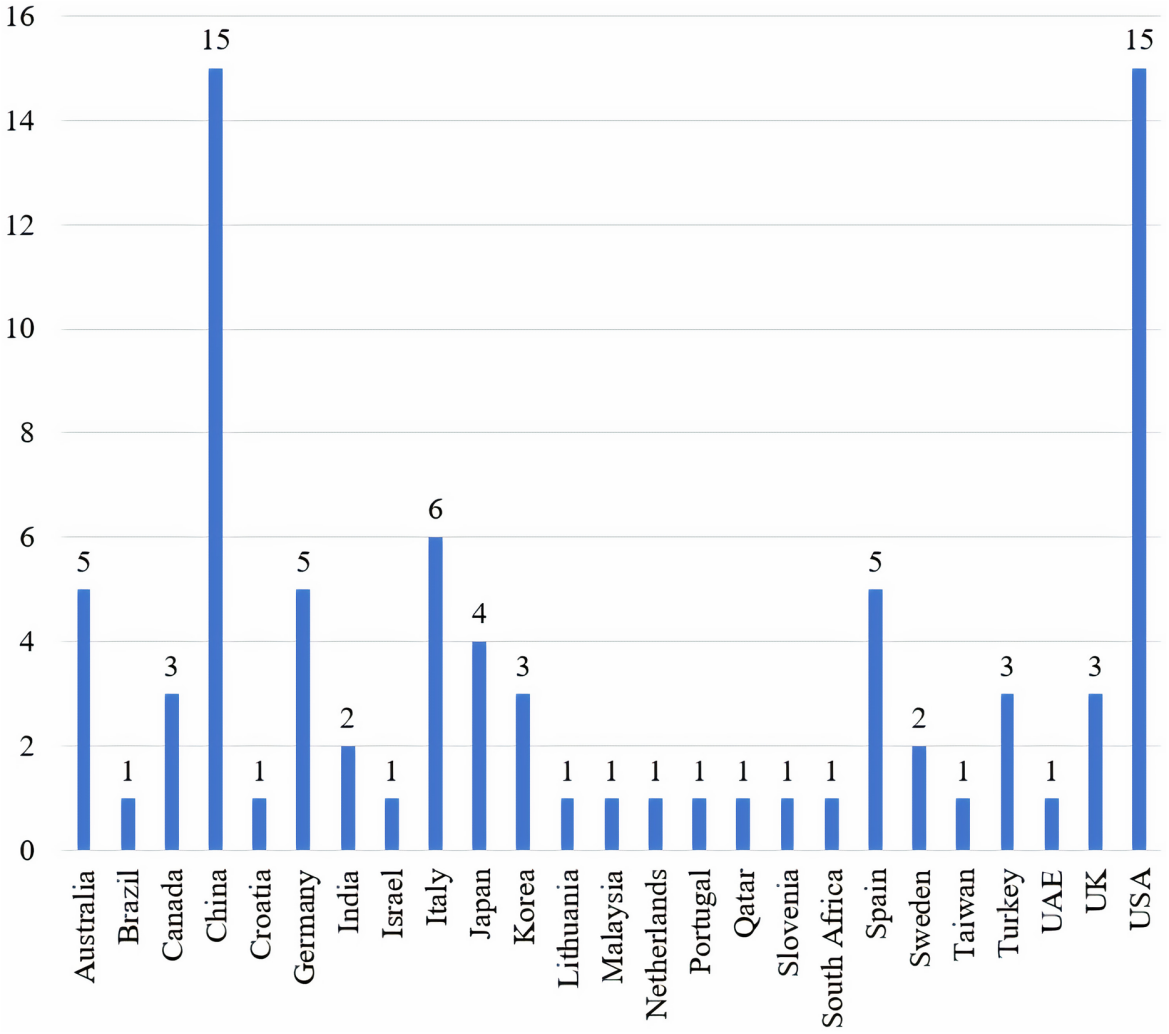
Distribution of articles with relation to authors’ affiliation.

Figure 7 shows the distribution of finalized articles according to the first authors’ affiliation. It is fair to state that the majority of countries, in which in-vehicle sensor based-experiments were conducted, are developed countries. Such a report should motivate researchers in countries with minimal or no reported studies. A model that compares drivers’ behaviour of multiple groups of people in different countries is a research gap in the current literature that needs to be further explored.

### Real-time experiments as a data source

During the filtration process, studies that rely on questionnaires, interviews, or simulations as data sources are excluded. Moreover, studies that rely on existing NDS data are also excluded, leaving only studies that generate their datasets, based on real-time experiments, in the final set of filtered articles. Table 3 lists those studies along with information related to participating samples, number of vehicles used, and experiments’ settings. Table 4 lists the sensors used in those experiments and the collected data.

**Table 3:** Information related to real-time experimental studies.

Ref.	Drivers	Age group (in years)	Male drivers	Female drivers	Driving experience (in years)	Car	Experiments’ settings (type of roads, time of experiments, duration of experiments)
Carmona et al. (2015)	10	N/A	N/A	N/A	N/A	N/A	Type: urban, inter-urban. Duration: 2 scenarios, 10 min each
Derbel (2018)	4	50, 50, 50, 70	N/A	N/A	N/A	N/A	Type: residential area, highway
Cai et al. (2018)	25	22–57	N/A	N/A	2–18	1	Type: urban
Park et al. (2016)	80	N/A	N/A	N/A	< 2	1	Test drive road
Khandakar et al. (2019)	N/A	N/A	N/A	N/A	N/A	1	N/A
Bastos et al. (2020)	6	19–38	4	2	<1, 4, 7, 10, 21	6	Type: total 1,303 km. Duration: 58.38 h
Schakel et al. (2017)	8	40–49 50–59, 60+	7	1	10–19, 20+	8	Type: freeway. Duration: 48 h
Varotto et al. (2017)	23	25–51 (avg 31.57)	15	8	3–12, 13–33	1	Type: 35.5 km freeway. Time: 7–9, 16–18, 18–20 Duration: 11 days
Varotto et al. (2018)	23	N/A	15	8	N/A	1	Type: 46-km, freeway. Time: 7–9, 16–18, 18–20. Duration: each driver 45–90 min
Galarza & Paradells (2018)	Two tests (20 & 15)	25–40, 21–35	N/A	N/A	>=30,000 km	N/A	Type: route was 38 km. Duration: avg. 70 min for drivers
Tonguç, Akçay & Gürbüz (2018)	N/A	N/A	N/A	N/A	N/A	1	Type: smooth asphalt road, damaged asphalt road, damaged lane road
Fridman et al. (2016)	1	N/A	1	0	N/A	1	Duration: 5 runs, duration of each run 37–68 min
Xiao et al. (2020)	N/A	N/A	N/A	N/A	N/A	1	Urban
Kim & Baek (2020)	N/A	N/A	N/A	N/A	N/A	10	Type: 5 km road. Duration: each vehicle was driven 3 times over the predestined road
Bender et al. (2015)	1	N/A	N/A	N/A	N/A	1	Type: suburb. Duration: 13 min
Andria et al. (2016)	2	N/A	N/A	N/A	N/A	1	Type: urban
Li et al. (2018)	10	N/A	N/A	N/A	N/A	N/A	Type: city-urban. Time: 9–11, 15–17. Duration: 16 h
Büyükyildiz et al. (2017)	1^st^ test 67, 2^nd^ test 51	18–20, 21–26, >26	N/A	N/A	1^st^ 80000 km, 2^nd^ 25	1	1st test highway, 2nd test urban and highway
Liu et al. (2017)	1	N/A	N/A	N/A	N/A	1	Type: circuit routes
Chen et al. (2019)	124	N/A	N/A	N/A	N/A	124	Type: city of Beijing. Time: 7–12. Duration: each driver drove approx. 480 h
Bichicchi et al. (2020)	10	N/A	N/A	N/A	N/A	1	Type: circuit route, 2,500 m. time: morning, afternoon
Taniguchi et al. (2014)	1	N/A	N/A	N/A	N/A	1	Type: circuit routes
Wu & Jovanis (2016)	76	20–70 (avg 41.6)	47.8%	53.3%	<10, 10–30, >30	11	Type: freeway, major arterial, minor arterial Time: daylight. Duration: each driver 4 weeks
Bella & Nobili (2020)	16	23–45 (avg 31)	N/A	N/A	>4	1	Type: 3.5 km route, 11 non-signalized and 4 signalized intersections. Time: Peak-off hours
Zylius (2017)	N/A	N/A	N/A	N/A	N/A	1	N/A
Wu, Chen & Yeh (2013)	3		N/A	N/A	N/A	1	Type: freeway, highway, suburb
Gündüz et al. (2017)	N/A	N/A	N/A	N/A	N/A	252	Duration: over 10 months
Liu & Hansen (2019)	20 in 2017, 13 in 2018	N/A	N/A	N/A	experience, novice drivers	1	Type: 2–3 lanes, local road. Time: Summer, sunny, daytime Duration: 3 rounds, 20 min each
Zheng et al. (2017)	15	<25, 25–35, 35–45, 45–55, >60	8	7	min 1year experience	N/A	Type: 2 routes in Florida University Campus Time: 16:40. Duration: Sept-Dec 2013
Yuksel & Atmaca (2020)	3	27, 28, 29	3	0	8–10	3	Type: urban. Time: Sunny. Duration: 2 weeks
Silva & Eugenio Naranjo (2020)	50	25–34, 35–54, 55–65 (avg 48.24)	31	19	avg 15.78	1	Type: 8 km, urban highways, suburban. Time: daytime. Duration: both routes had duration of 24 min
Lee & Jang (2017)	43	N/A	N/A	N/A	N/A	N/A	Type: metropolitan areas. Duration: 8 days
Li et al. (2015)	78	N/A	N/A	N/A	N/A	N/A	Type: urban highway. Duration: avg 105 min per driver
González et al. (2014)	10	27–48	10	0	N/A	N/A	Type: road network. Duration: 2 days
Joubert, Beer & Koker (2016)	N/A	N/A	N/A	N/A	N/A	124	Duration: 1 month
Zardosht, Beauchemin & Bauer (2018)	12	20–47	6	6	N/A	N/A	Type: urban, 28.5 km. Time: daytime. Duration: 1 h
Hong et al. (2016a)	1	N/A	1	0	expert driver	1	Type: snowy road, asphalt road, local road
Yao et al. (2020)	120	N/A	N/A	N/A	N/A	120	N/A
Krasniuk et al. (2019)	35	18–59 (avg 50)	14	21	N/A	1	Type: parking lot, suburban, highway
McLaurin et al. (2018)	100 (66 OSA, 34 compare)	N/A	62 (44 OSA, 18 comparison)	38 (22 OSA, 16 comparison)	N/A	100	Type: two-week period before and three months after beginning CPAP
Svetina (2016)	351	20–35, 35–50, 50–65, 65–80	N/A	N/A	min 2 years	N/A	Type: practice track 300 m
Merickel et al. (2019)	77	65–90	41	36	40–76 (avg 58)	77	Type: rural, urban. Time: daytime, nighttime Duration: 2–3 months periods separated by 1 year
Farah et al. (2014)	217 families	N/A	N/A	N/A	N/A	N/A	Duration: 11 months
Gadepally, Krishnamurthy & Ozguner (2013)	N/A	N/A	N/A	N/A	N/A	1	Type: highway, urban. Duration: 1 h
Dabbour (2015)	28	19–73 (avg 40.5)	15	13	N/A	28	Duration: 48 h
Yi et al. (2018)	3	N/A	N/A	N/A	N/A	N/A	Duration: 225 min
Lubbe & Rosén (2014)	62	20–61 (avg 42)	58%	42%	3–41 (avg 23)	N/A	Type: urban
Silva et al. (2014)	5	40–55	3	2	20	1	Type: 3.6 km arterial urban road
Zyner, Worrall & Nebot (2019)	N/A	N/A	N/A	N/A	N/A	1	Type: 5 roundabout in suburbs, high traffic Time: Morning, afternoon. Duration: each recording of roundabout was 14 h
Li et al. (2019)	20	avg 41.7	16	4	16.8	3	Type: urban
Cerni & Bassani (2017)	21	20–50	17	4	N/A	N/A	Type: two-lane rural highways
Montella et al. (2014)	39	23–70 (avg 35.77)	25	14	min. 5,000 km in year	1	Type: rural motorway. Time: 9:30 AM–4:30 PM on workdays. Duration: 2 months
Satzoda & Trivedi (2014)	2	N/A	N/A	N/A	N/A	1	Type: highway
Gao, Murphey & Zhu (2018)	10	N/A	5	5	novice to expert	N/A	Type: 2–3 lanes, local and freeway. Time: daytime, sunny, cloudy. Duration: 20 trips, 10–40 min each
Xie et al. (2018)	50	N/A	N/A	N/A	N/A	N/A	Type: highways, ring road, airport express, normal city road (18km)
Hill, Elefteriadou & Kondyli (2014)	1st test 15, 2nd test 31	30–39	1st test 6/2nd test 19	1st test 9/2nd test 12	N/A	N/A	Type: freeway
Zhao et al. (2016), Yao et al. (2016)	1	N/A	N/A	N/A	N/A	1	Type: expressway, 64 km
Gao, Murphey & Zhu (2019)	10	18–40	5	5	novice-to-expert	N/A	Type: 2–3 lanes, local and freeway. Time: daytime, sunny, cloudy. Duration: 22 trips, 10–40 min each
Wang et al. (2019)	15	27–48 (avg 34.7)	13	2	3–23 (avg 8.4), 3 year no accident	1	Type: expressway
Jermakian et al. (2017)	40	16-17	20	20	6-9 months	1	Type: highway, urban, suburban. Duration: 14 months (3,259 h)
Llorca, Moreno & Garcia (2016)	N/A	N/A	N/A	N/A	N/A	1	Type: rural
Katzourakis et al. (2013)	8	N/A	6	2	N/A	1	Duration: each driver had 16 runs
Hassan et al. (2017)	4	26, 28, 33, 33	N/A	N/A	7, 8, 13, 10	N/A	Type: two-sided straight road, width 3.5 m
Heesen et al. (2014)	45	23–50 (avg 30.4)	27	18	Min. 1 year	1	Type: test track, 2 km. Duration: 1 h each driver
Hildebrandt et al. (2015)	63	N/A	54%	46%	N/A	1	N/A
Butakov & Ioannou (2014)	3	26, 27, 30	3	0	3, 4, 5	1	Duration: 6–18, 4, 3 h
Bifulco et al. (2014)	100	(20–24), (25–40) (41–64), >65	N/A	N/A	N/A	N/A	Type: highway, urban. Time: 8:30–18:30. Duration: 120 h
Schorr, Hamdar & Silverstein (2014)	18	20–33	9	9	N/A	1	Type: predefined loop featured 4 segments
Errampalli, Mallela & Chandra (2020)	1	N/A	N/A	N/A	N/A	1	Type: 3 km urban corridors and 25 km non-urban corridors. Duration: 3 runs on urban corridors and 3 runs on non-urban corridors
Das et al. (2019)	2	N/A	N/A	N/A	N/A	2	Type: 37.33 km, rural. Time: 14:00–16:30
Qi et al. (2015)	10	N/A	N/A	N/A	N/A	1	Type: urban. Time: 8–9, 9–10. Duration 2 h each driver
Wang et al. (2014)	36	30–69 (avg 44.9)	N/A	N/A	1–47 (avg 12)	1	Type: urban, Freeway
Peng et al. (2019)	51	25–56 (avg 37)	45	6	3–16 (avg 12)	1	Type: urban. Time: 7:30–9:30, 17–19. Duration: 265.8
Stigson et al. (2014)	196 (128 test, 68 control)	22–66	65.6%	34.5%	N/A	N/A	Duration: 11 months
Stephens et al. (2015)	19	22–47 (avg 30.84)	12	7	2-30 (avg 12.78)	1	Type: freeway, arterial road. Duration: 50 min
Hong et al. (2016b)	20	22–24 (avg 23), 69–78	7 young, 5 old	3 young, 5 old	1y (young drivers)	N/A	N/A
Turner, Woolley & Cairney (2015)	40	N/A	40	0	<3, >=15	1	Type: rural driving route. Duration:1 h:35 min each driver
Maljković & Cvitanić (2016)	20	25–60	13	7	2–30	20	Type: 24 km long segment. Time: Daylight Duration: Oct 2012–April 2013
Fleming et al. (2019)	9	25–40	N/A	N/A	Min. 3 years	N/A	Duration: 7 h for car following scenarios and 10 h for cornering scenarios
Wang et al. (2016)	24	N/A	20	4	N/A	3	Type: urban
Lyu et al. (2019)	32	22–55 (avg 32.2)	21	11	2–18 (avg 6.9)	N/A	Type: urban, expressway, freeway. Time: 8:00–17:30. Duration: 6,500 km of data
Salmon et al. (2017)	20	25–49 (avg 38.3)	10	10	avg 20.6	1	Type: 15 km urban route in the suburbs

**Table 4:** Sensors utilized in reported experiments and their correspoding recorded data.

References	CAN	OBDII	IMU	Accelerometer	Gyroscope	Lidar	Radar	Pedal sensor	Laser	Camera	MobileEye	GPS	Recorded data for analysis (A, acceleration; B, braking; D, deceleration; DI, distance; LA, lateral acceleration; RPM, revolutions per minute; S, speed; SA, steering angle; THW, time headway; TTC, time to collision; VA, vertical acceleration; VHW, vehicle headway; Y, yaw)
Carmona et al. (2015)	✓			✓	✓			✓				✓	S, A, B, RPM, LA, SA
Derbel (2018)		✓		✓								✓	S, A
Cai et al. (2018)	✓			✓				✓		✓	✓		S, A, B, LA, Y, SA
Park et al. (2016)	✓							✓				✓	S, A, D, B, RPM, C, SA
Khandakar et al. (2019)		✓		✓									S, A, B, RPM
Bastos et al. (2020)										✓		✓	S
Schakel et al. (2017)	✓	✓								✓			S, A, D, THW, VHW
Varotto et al. (2017)							✓	✓				✓	S, A, D, B, THW, VHW, DI
Varotto et al. (2018)	✓						✓	✓				✓	S, A, D, B, TTCi, VHW, DI
Galarza & Paradells (2018)	✓									✓			S, A, B, LA, SA
Tonguç, Akçay & Gürbüz (2018)		✓		✓				✓				✓	S, A, B, LA, VA, SA
Fridman et al. (2016)	✓		✓	✓						✓		✓	S, A, SA
Xiao et al. (2020)		✓										✓	S, SA
Kim & Baek (2020)	✓		✓							✓		✓	S, A, D, B, SA
Bender et al. (2015)			✓	✓	✓								S, A, D, B, Y
Andria et al. (2016)		✓	✓	✓	✓				✓			✓	S, A, D, B, RPM, C
Li et al. (2018)				✓									S, A, LA, VA
Büyükyildiz et al. (2017)	✓						✓			✓			S, A, D, LA
Liu et al. (2017)	✓							✓					S, A, B, LA, Y, SA
Chen et al. (2019)		✓										✓	S, A, D, RPM
Bichicchi et al. (2020)	✓									✓		✓	S, A, LA, A
Taniguchi et al. (2014)	✓							✓		✓			S, A, B, TTC, VHW, SA
Wu & Jovanis (2016)												✓	S
Bella & Nobili (2020)										✓		✓	S, DI, D, TTC
Zylius (2017)					✓								S, A, LA, VA
Wu, Chen & Yeh (2013)			✓	✓						✓		✓	S, A, D, LA, DI
Gündüz et al. (2017)				✓								✓	S, A, D, LA, SA
Liu & Hansen (2019)		✓				✓				✓		✓	S, A, D, B, SA, DI
Zheng et al. (2017)										✓		✓	S, B
Yuksel & Atmaca (2020)			✓										A, D, SA
Silva & Eugenio Naranjo (2020)		✓											S, A, D, B
Lee & Jang (2017)		✓											S, A, D, Y, SA
Li et al. (2015)												✓	S, A, D, B, LA
González et al. (2014)												✓	S, A, LA
Joubert, Beer & Koker (2016)				✓								✓	S, A, LA, VA
Zardosht, Beauchemin & Bauer (2018)	✓	✓						✓		✓		✓	S, A, B, DI
Hong et al. (2016a)	✓			✓	✓								S, Y, LA, SA
Yao et al. (2020)		✓										✓	S, A, D, Y
Krasniuk et al. (2019)												✓	S, DI
McLaurin et al. (2018)		✓		✓						✓		✓	S, A, LA
Svetina (2016)				✓						✓			D
Merickel et al. (2019)		✓		✓						✓		✓	S, A, D, B, SA
Gadepally, Krishnamurthy & Ozguner (2013)	✓									✓		✓	S, A, RPM, LA, Y
Dabbour (2015)												✓	S, A
Yi et al. (2018)			✓	✓								✓	S, A
Lubbe & Rosén (2014)								✓				✓	S, A, D, B, TTC, THW, DI
Silva et al. (2014)				✓								✓	S, A, LA
Zyner, Worrall & Nebot (2019)						✓							DI
Li et al. (2019)				✓								✓	S, A, LA
Cerni & Bassani (2017)												✓	S, SA
Montella et al. (2014)	✓				✓		✓	✓		✓		✓	S, A, D, B, C, VHW, SA
Satzoda & Trivedi (2014)	✓			✓				✓		✓		✓	S, A, B, Y, SA
Gao, Murphey & Zhu (2018)		✓								✓			S, SA
Xie et al. (2018)	✓					✓				✓			S, LA, SA
Hill, Elefteriadou & Kondyli (2014)										✓		✓	S, A, B
Zhao et al. (2016), Yao et al. (2016)	✓		✓	✓	✓	✓						✓	S, A, Y
Gao, Murphey & Zhu (2019)	✓	✓								✓		✓	S, DI, SA
Wang et al. (2019)	✓						✓			✓		✓	S, D, A, DI
Jermakian et al. (2017)	✓			✓			✓			✓		✓	S, A, THW, DI, Y
Llorca, Moreno & Garcia (2016)									✓	✓		✓	S, A, D, DI
Katzourakis et al. (2013)	✓		✓	✓	✓							✓	S, A, LA, SA
Hassan et al. (2017)	✓		✓				✓						S, TTC, DI, Y
Bifulco et al. (2014)							✓			✓		✓	S, A, TTC, THW, DI
Schorr, Hamdar & Silverstein (2014)		✓				✓						✓	S, A, THW, VHW
Errampalli, Mallela & Chandra (2020)										✓		✓	S, A, D, DI
Das et al. (2019)										✓		✓	S, TTC, DI
Qi et al. (2015)							✓			✓		✓	S, A, DI
Wang et al. (2014)							✓	✓		✓		✓	S, A, B, TTC, TTCi, THW, Y, SA
Peng et al. (2019)	✓						✓	✓		✓	✓	✓	S, A, D, B, TTC, VHW, SA
Stigson et al. (2014)												✓	S
Stephens et al. (2015)								✓		✓		✓	S, A, D, B, SA
Hong et al. (2016b)								✓		✓		✓	S, A, D, B, LA
Turner, Woolley & Cairney (2015)				✓	✓					✓	✓		S, A, D
Maljković & Cvitanić (2016)												✓	S, A
Fleming et al. (2019)										✓		✓	S, A, TTC, TTCi, THW, VHW, LA, DI
Wang et al. (2016)	✓							✓				✓	S, A, B, TTC, TTCi, LA
Lyu et al. (2019)	✓			✓		✓		✓		✓	✓		S, A, D, B, THW, VHW, LA, SA
Salmon et al. (2017)	✓			✓						✓		✓	S, A, B, SA

Researchers have used several sensors to collect data in real-time experiments and generate their respective datasets. Studies that have not specified the sensors used in their experiments are excluded from Table 4. Ideally, researchers use devices/sensors such as CAN-bus and OBDII to record vehicle speed; IMU, gyroscopes, and accelerometers to record steering behaviour; GPS to record vehicle position; lidar and radar to record distances between the experimental vehicles and the preceding vehicle. Some researchers, however, use some sensors for more than one purpose, such as using GPS not only to record vehicle position but also to record vehicle’s speed and steering behaviour. Such a technique is sometimes adopted by researchers to reduce the number of sensors/devices used in their proposed DAS, thus reducing its cost. The literature analysis indicates the need for a reliable low-cost DAS. Currently, there is no study, in the current literature, that provides clear guidelines on how to build a reliable cost-efficient DAS, nor a study that specifies which combination of sensors is most suitable to use than others when building DAS.

### Sample size

**Figure 8:**
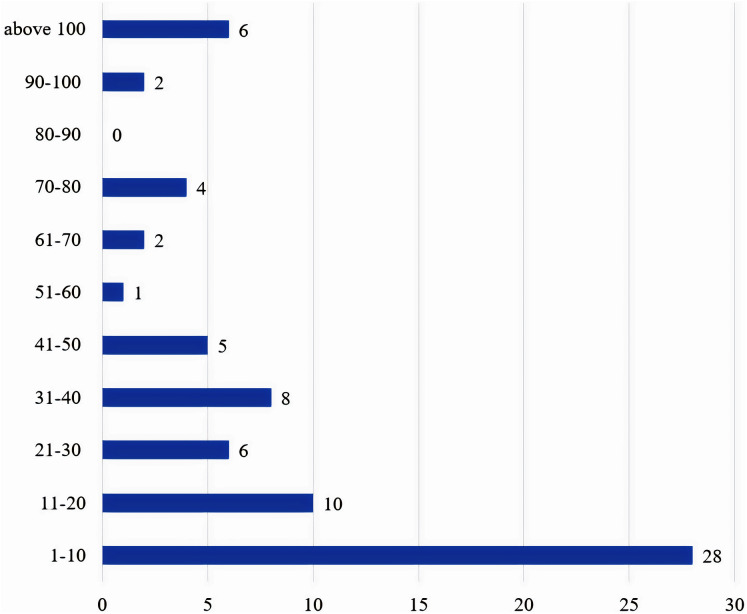
Distribution of sample sizes across the filtered articles.

Table 3 lists the number of participating drivers, reported in the articles reviewed. The articles that have not reported the number of participating drivers are excluded from this analysis. Due to the variable number of sample sizes reported, the authors categorize them into 11 groups as seen in Fig. 8.

A total of 72 studies have reported the number of drivers employed in their experiments. About 39% use a sample size of no more than 10 drivers. Almost 53% of studies use a sample size of 20 drivers at the most. This analysis concludes that when researchers do real-time experiments, most of them use very limited sample sizes. The authors recommend that future researchers utilize a sample size of at least 30 drivers to generalize their findings and ensure their results are not ‘sample size dependent’.

### Number of vehicles used in experiments

**Figure 9:**
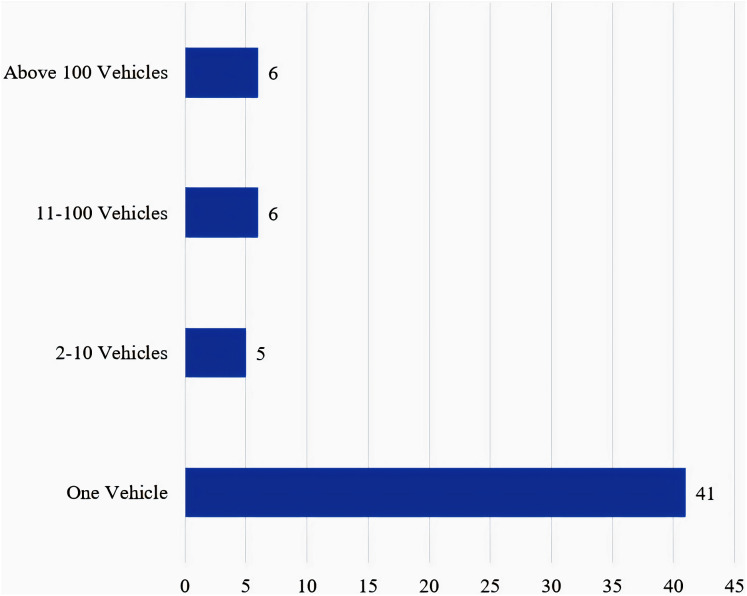
Reported number of vehicles used in the filtered literature.

Most researchers have utilized one vehicle in their experiments for data collection, few studies have utilized more than one vehicle. The authors are careful not to assume the number of utilized vehicles unless it is stated in the articles. Table 3 lists the number of vehicles utilized in the reviewed articles. A total of 58 studies reported the number of vehicles utilized in their research, and out of those studies, 70% (41 studies) use a single vehicle. This limited number of vehicles can lead to limited representation of data, given that one vehicle cannot represent the complete picture of driving behaviours. Furthermore, the use of a limited number of vehicles prolongs the experiments’ duration, hence drivers must await their turn in a queue. However, increasing the number of vehicles is also challenging, because it increases the cost of experiments’ design and DAS installation. Also, installing the DAS in participants’ vehicles, to include more vehicles during experiments, makes the collected data susceptible to biases because not all drivers drive the same vehicles. Figure 9 presents the number of vehicles used in the reported experiment of the reviewed articles. The authors recommend that future researchers allow all participating drivers to drive at least two vehicles, to enable more representation of vehicles and to answer a gap in the current literature. This gap is presented in the form of two questions: does changing the vehicle affect driver’s behaviour? and if it does, does the effect of changing vehicles become statistically significant on drivers’ behaviour?

### Males to females ratio in participating samples

The number of males and females who participated in real-time experiments have been reported in Table 3. Of those who reported the ratio of male to female drivers, 32 studies have reported using more male drivers than female drivers, 6 studies used the same ratio of male drivers and female drivers, and only 2 studies reported using more female drivers than male drivers. This survey analysis concludes that most studies (80%) use more male drivers than female drivers and are unsuccessful in considering gender variation in their reported samples, as female drivers are currently under-represented in the collected driving datasets; which makes the inclusion of more female drivers a necessity in potential future researches. The authors recommend that future researchers collect data from an equal number of male and female drivers to ensure their sample is equally diverse in its gender representation and to identify the major differences in driving behaviour of male and female drivers.

### Drivers’ age

Drivers of various ages (18–90) have participated in previous studies, as seen in Table 3, however, studies are limited in their use of samples aged above 60 or below 20, since most studies concentrated on sampling the majority of drivers within the age range of 25 to 50 years old. This makes the representation of adolescent drivers and elder drivers very limited in the collected datasets. The authors recommend that future researchers include drivers of ages above 55 years old and below 22 years old to ensure their sample is diverse in age representation. Also, to identify the root causes of differences in driving behaviour between drivers of various age ranges.

### Drivers’ experience

There are various metrics, used in the literature, for determining drivers’ experience. The first metric is the years of driving, which is concluded from the year on which the drivers’ licenses are issued. The second metric is the total amount of distance traveled by the driver since he started driving. The third metric is the number of times per month the driver usually drives. There are other notions used by previous researchers to describe drivers’ experience, such as the term ‘experience vs inexperience’; such metrics are very subjective since they can have a different interpretation for different people. Diversity in utilizing drivers of variable experiences is important in future studies because novice drivers are disregarded in most experimental studies in present-day literature. Table 3 shows the experience level of drivers reported in the literature. The authors recommend avoiding notions that are subjective, such as the second and third metric, as both can be difficult to accurately estimate, and rely on the first metric when reporting drivers’ experience.

### Experiments settings (route, time and duration of experiments)

This section analyzes the information related to the road type, time, and duration of reported experiments. Table 3 lists the aforementioned information reported in the reviewed articles. For route of experiments, it is difficult to conclude the most commonly used routes. This is because not all studies used the same terms to describe route types and that some studies allowed drivers to drive using their vehicles for weeks or months in a city, which makes it difficult to pinpoint which routes the drivers drove on. However, it is safe to say that experiments are conducted on driving areas that can be described as urban, suburban, rural, expressway, freeway, and highway. Some researchers stated only the length of the road. Some researchers stated only the shape of the road, such as circuit road, ring road, minor arterial road, and major arterial road. Some experiments are conducted at parking lots and university campuses. There is a limited number of studies conducted in rural areas, as seen in Table 3, which could serve as a motivation for future researchers. Also, results accumulated from experiments that are performed on private fields and university campuses may have disputed validity when applied to other routes. Researchers should aim to conduct experiments on various roads. However, this would increase the overall cost and duration of experiments, which could be challenging for researchers with limited funds.

For the time of experiments, some articles specified the exact periods on which the experiments are conducted, such as at ‘8:00 AM to 12 PM’. Few articles use terms such as ‘daytime’ or ‘daylight’ while others use terms such as ‘weekends’ or ‘workdays. A research that compares driving behaviour in rush hours and driving behaviour in normal hours seems necessary.

For the duration of experiments, some studies took as little as 13 min, while others as much as 480 h for each driver. The duration of experiments is correlated to the objective of the study. If the study aims to collect naturalistic data using a DAS installed in the drivers’ vehicles, then usually the experiments’ duration is somewhat lengthy; however, if the study aims to examine driver behaviour with relation to a specific aspect, such as braking behaviour, then the duration of experiments is usually short. Some studies prefer to mention the total distance traveled by drivers during experiments rather than the actual duration, such notions are often stated in studies that installed the DAS in the drivers’ vehicle.

Most researchers conducted their experiments in sunny/cloudy conditions. It is important to note that the use of the terms ‘good’ or ‘bad’ weather conditions, in the reported literature, are somehow subjective as they have different interpretations for different individuals. A research that considers the effect of variable temperatures and visibility ranges on driver behaviour is deemed necessary; such metrics are seldom cited in publications.

In summary, the authors recommend that future researchers select a suitable route, that contains various road types, for their experiments. For instance, a route that contains highways, intersections, part of it is urban and the other part is rural. It is also recommended that researchers perform the experiments during the same periods each day under the same weather conditions so that external factors such as weather, visibility, and traffic would not affect the data collection process and distort future measurements and analysis; unless the study is purposely set to analyze the differences of driving in various weather, visibility and traffic conditions.

## Future research directions

Based on the authors’ comprehension of the available literature, general directions are presented in this section to help future researchers who are interested in driver profiling and recognition. Several key points are addressed for future improvements, including building efficient DAS, establishing reliable and accurate driver profiles, modulating and evaluating deep learning-based recognition systems that can classify drivers according to their established profiles.

### Phase one: sensors selection, installation and verification for DAS

To collect driving data from real-time experiments, researchers should build their own DAS. The DAS must be built according to the data that the researchers aim to record. Different studies record different data for analysis. For example, in a car-following context, distances between the ego vehicle and the leading vehicle are very important; therefore, distance sensors, such as lidars or radars, must be used. Also, in lane changing context, the rotational movement of the vehicle is crucial, therefore, the use of an accelerometer, a gyroscope, or both (IMU) is essential. For studies that aim to capture overall driver behaviour, multiple sensors should be used to record the speed, distance, and steerings, such as OBDII, lidar or radar, and gyroscope or IMU. The choice of which sensors to assemble in the DAS can be affected by factors, such as cost and performance. For example, some researchers prefer to use smartphone sensors and GPS to record driving data; however, smartphone sensors usually require high-performance computational capabilities (Khandakar et al., 2019) and have overestimation issues (Joubert, Beer & Koker, 2016) and GPS has similar issues. Also, 3D lidars are considered highly accurate, but they are considerably costly (Zhao et al., 2016). A substantial cost could be required for constructing and developing DASs, especially when the study aims to collect naturalistic driving data from multiple vehicles. Thus, building a reliable, low-cost DAS is a tremendous step towards research advancements. Hence, certain specifications and constraints must be set to achieve high-quality results, including:

- Proposed DAS should be safe to install with no exposed wiring carrying electrical currents that might harm the drivers.
- Proposed DAS should be easy to install inside the vehicle. It should be as non-visible as possible, to ensure that it does not influence the naturalistic driving behaviour of drivers.
- Each sensor in the proposed DAS must be verified by comparing its readings with the readings of a secondary sensor. This process is important to ensure that the main sensors of the proposed DAS have no manufacturing defects. For instance, to validate the OBDII, a second sensor such as GPS is set to record speed data, and then the collected speed data of the OBDII and GPS are compared to each other. Even though GPS is not as accurate as an OBDII, the simultaneous increase and decrease of speed data in both devices proves that the OBDII is operating correctly and has no manufacturing defects. The same technique should be used to verify all sensors in the proposed DAS.
- The proposed DAS should be adaptable for integration with other sensors and gadgets in the future.

### Phase two: data collection

To appropriately set the data collection phase, several factors must be identified:

- Most studies in the literature utilize a limited sample size between 1 and 20, the authors recommend using a sample of 30 drivers or above, to ensure the collected data is not ‘sample size dependent’ and therefore results are not biased and can be generalized.
- Participating drivers should be of age between 20 and 60 years and are divided into four groups, distributed as follows: group A includes drivers within the age range of 20–30 years old, group B includes drivers within the age range of 31–40 years old, group C includes drivers within the age range of 41–50 years old and group D includes drivers within the age range of 51–60 years old. Such grouping would be useful in providing an in-depth analysis of the effect of age on driving performance (young *versus* older drivers).
- Female drivers are very underrepresented in the current literature, the authors recommend using a sample size of 50% men and 50% women. Results from such settings can provide valuable insights on differences between men’s driver behaviour and women’s driver behaviour (male *versus* female drivers).
- Participating drivers must carry a driving license covering a minimum of one-year validity, this is to ensure that participating drivers have the required driving knowledge to perform experiments with minimal risk.
- Experiments carried on routes containing multiple geometric locations, such as highways and intersections, are preferable. Such data can be used to provide valuable information on how drivers’ behaviour changes with relation to different road types.
- Experiments should be carried out on the same periods under similar weather conditions; this is to ensure that all participating drivers drive under the same conditions, therefore ensuring consistency in data collection and eliminating external factors that could skew the future analysis. Unless the study is purposely set to model driving in various weather, visibility, or traffic conditions.
- A recognition system that adapts current deep learning algorithms is presented in phase 4. Neural networks generally require large datasets for training; therefore, the authors suggest that experiments’ duration for each participating driver would be a minimum of 20 minutes, to ensure enough data is recorded for the training of the recognition system. Therefore, the route’s length should be planned accordingly by researchers.

### Phase three: driver profiling

After data collection, it is recommended to label drivers according to their safe/aggressive behaviour, this is called driver profiling. Through this labeling process, the driver behaviour aggressive scale is constructed, which is a scale on which drivers are classified into different levels of behaviours. Too often, researchers label drivers according to questionnaires filled by the drivers themselves or by relying on existing profiles from previously published articles. However, such techniques are not advisable, because asking the drivers if they are aggressive can be biased and subjective Lee & Jang (2017) and Qi et al. (2015). Also, most previously published articles either rely on questionnaires as well or used similar biased labeling techniques, such as observations and crash reports. Furthermore, what is considered “aggressive behaviour” in one state or country could be different in another country. For instance, collecting NDS data from Malaysian drivers in Kuala Lumpur and then label them according to publications from the US presents many issues; as traffic laws and regulations are different in these two countries, and what is considered as “aggressive behaviour” in Malaysia could be different from the US. Therefore, researchers should aim to profile drivers based on the traffic laws and regulations of their respective countries, to avoid labeling drivers as safe when they are aggressive in the eyes of the law and vice versa. The authors recommend the labeling process be done by experts who have considerable knowledge of the laws and traffic regulations in the country in which the experiments are conducted. Such a technique would establish driver profiles associated with the country/state traffic laws and regulations. Very few studies aim to do this technique. For example, in reference (Zylius, 2017), in which Lithuanian experts label the drivers into two categories (aggressive and safe). Another example is in reference (Gündüz et al., 2017), which categorizes aggressiveness into three groups, low risk, medium risk, and high risk, based on legal authority reports in Turkey. Also, in another study in Turkey, in reference (Yuksel & Atmaca, 2020), where traffic officers evaluate risky driving behaviours, the risky behaviours are given a score between 1 and 10. Also, in reference (Lee & Jang, 2017), which categorizes drivers into three levels of aggressiveness (high, medium, low) according to the Korean roadway operation guidelines. Moreover, in reference (Silva & Eugenio Naranjo, 2020), where experts from Spain evaluate driving tests by categorizing each driver as calm, normal, or aggressive. Furthermore, in reference (Li et al., 2015), where risk consulting experts in Japan assign aggressiveness scores, and through these different scores (from 1 to 5, where 1 being least aggressive and 5 being the most aggressive), the driver behaviour aggressive scale is constructed. The authors recommend that future researchers follow experts’ guidance and directions when labeling and profiling drivers. Table 5 summarizes the aforementioned studies.

**Table 5:** Studies in which experts profilied drivers.

Reference	Year	Levels in the driver behaviour aggressive scale	How labelling is done
Zylius (2017)	2017	2 levels (aggressive, safe)	Labelling done by experts from Lithuania
Gündüz et al. (2017)	2017	3 levels (low, medium, high)	Labelling done by experts from Turkey
Yuksel & Atmaca (2020)	2020	1–10 risk level score (where 1 is very low risk and 10 is very high risk)	Risk levels were evaluated in accordance with the expert opinions of traffic officers in Turkey
Silva & Eugenio Naranjo (2020)	2020	3 levels (calm, normal, aggressive)	Evaluation and categorization of drivers was done by experts in Spain
Lee & Jang (2017)	2017	3 levels (low, medium, high)	Labelling was based on Korean Roadway Operation Guidelines
Li et al. (2015)	2015	1 to 5 (1 is least aggressive and 5 is most)	Labelling done by experts from Japan

### Phase four: deep learning-based recognition system

In previous phases, the researchers have developed DAS, collected experimental data from participating drivers, and profiled the drivers. This phase aims to develop and modulate a driver behaviour recognition system that would classify drivers, according to the driver behaviour aggressive scale, using deep learning methods. The reason for using this approach instead of other approaches (like rule-based approaches) is because deep learning is an alternative approach that can help address some of the issues with traditional methods. Rather than attempting to fully emulate the decision process of the experts, deep learning algorithms typically only take the outcomes from the experts. For example, the expert may review several driving scenarios and decide which are aggressive and which are not. Exactly how the expert arrives at his decision is not important for the deep learning algorithm, only what his decision is. Focusing on the outcomes rather than the entire decision-making process can make deep learning methods more flexible and less susceptible to some of the problems encountered with rules-based systems. The authors recommend using deep learning-based algorithms, instead of machine learning algorithms, because they provide deeper analysis, and structure algorithms in layers, to create a neural network that can learn and make intelligent decisions on its own. Moreover, it is more efficient when the resultant dataset is huge, which can be challenging to train and model using traditional machine learning algorithms. Before training the classifier, it is important to use a method that can select or combine variables into features, to effectively reduce the amount of data that must be processed, while still accurately describing the original dataset. This is called data cleansing and feature extraction, and it is important because, during experiments, sensors may record data that are irrelevant to the research. For example, a DAS built to record steering data from an IMU sensor and speed data from an OBDII sensor may also record information related to engine and air temperature, which are most likely irrelevant to the research. This is why this phase is important, especially if DAS contains multiple sensors, as such irrelevant data would get immense and subsequently affect the performance of the recognition system. After data cleansing and feature extraction, researchers should train the recognition system and evaluate its performance. Various classifiers should be used during the development of the recognition system, such as convolutional neural networks (CNN), deep neural networks (DNN), artificial neural networks (ANN) and recurrent neural network (RNN). Part of the dataset is used to train those classifiers. Various performance metrics determine which deep-leaning method is to be recommended for the recognition system. Those performance metrics are discussed further on the next phase.

### Phase five: evaluation

In the last phase, researchers should aim to evaluate the performance of their proposed recognition system using a confusion matrix. Since drivers are profiled in phase 3, researchers can use part of the dataset to train the classifiers and measure their performance according to how they accurately profile the drivers in the untrained dataset. Researchers can use performance metrics, listed in Table 6, as the basis for evaluating the performance of the deep learning classifiers of the recognition system.

**Table 6:** Performance metrics.

Metric	Definition	Equation
Accuracy	The ratio of correctly predicted observation to the total observations	(True Positives (TP) + True Negatives (TN))/(Positives+ Negatives)
Recall (sensitivity)	The ratio of correctly predicted positive observations to the all observations in actual class	TP/(TP + False Negatives (FN))
Precision	The ratio of correctly predicted positive observations to the total predicted positive observations	TP/(TP+ False Positive (FP))
f-measure	The weighted average of Precision and Recall	2 × (Recall × Precision)/(Recall + Precision)
False Positive Rate (FPR)	The proportion of samples that test positive which are genuinely negative	FP/(FP+TN)
False Negative Rate (FNR)	The proportion of samples that test negative which are genuinely positive	FP/(FP+TN)

## Conclusions

This systematic review is beneficial to the research community and is a major step in summarizing the literature with regards to sensor-based driver behaviour domain.

Research efforts in this area are still ongoing and this survey aims to contribute to its understanding by reviewing its current research efforts. Four databases are selected for this purpose and articles are filtered according to a set of eligibility criteria. A taxonomy of the current literature is presented to facilitate the analysis and categorization of filtered articles. Discussions on the motivations that drive researchers into this domain are stated, common issues and challenges are highlighted, and recommendations are provided. Substantial analysis of the filtered articles is presented to fill certain gaps in the literature. Furthermore, possible research directions, based on the authors’ understanding of the literature, are presented for future improvements. Those directions include guidelines for building an efficient DAS, suggestions on how to profile drivers according to their aggressive/safe behaviours, and the proposition of a deep learning-based recognition system that would classify drivers according to their established profiles.

For future work, the proposed five-phase methodology, in the future directions section, will be implemented. The design of the proposed DAS and the data collection process results will be made available for other researchers. Comparisons between various groups will be presented as well, such as comparisons between female drivers and male drivers, between young drivers and old drivers, between driving on weekdays and weekends, and between driving in normal traffic and driving in Covid-19 traffic restrictions. The performance of a deep learning-based recognition system that classifies drivers according to their behaviour will be presented and discussed.

## Additional Information and Declarations

### Data Availability

The following information was supplied regarding data availability: This article is a Systematic Literature Review without raw data or code.
